# Superior Division Oculomotor Nerve Palsy and Diabetes Mellitus: A Case Report

**DOI:** 10.7759/cureus.82612

**Published:** 2025-04-20

**Authors:** Konstantinos Flindris, Eleni Papafotiou, Elena Mylona, Chrysa Chatzipetrou, Athanasios Kaliardas, Ioannis Koumpoulis, Ioannis Melissourgos

**Affiliations:** 1 Ophthalmology, General Hospital of Ioannina "G. Hatzikosta", Ioannina, GRC

**Keywords:** diabetes mellitus, diabetic ophthalmoplegia, oculomotor nerve, oculomotor nerve palsy, superior division

## Abstract

Oculomotor nerve (third cranial nerve) palsy is a neuro-ophthalmic emergency that can signify life-threatening pathologies. Diabetes mellitus (DM) is a common cause of isolated oculomotor palsies. We report a case of an acute isolated ptosis and diplopia caused by a superior division oculomotor nerve palsy in a diabetic patient, highlighting the diagnostic approach and outcome. A 59-year-old man with type 2 DM and dyslipidemia presented with acute right upper eyelid drooping and binocular diplopia, without headache or other neurological symptoms. Examination revealed complete right-sided pupil-sparing blepharoptosis and inability to elevate the right eye, causing vertical diplopia on upward gaze. All other extraocular movements were intact. No other neurological deficits were present. Laboratory workup was unremarkable, except for a glycated hemoglobin (HbA1c) of 7.1%, indicating suboptimal glycemic control. Urgent neuroimaging of the brain and orbits was performed and showed no intracranial lesions. The findings were consistent with a superior division oculomotor nerve palsy due to diabetic microvascular ischemia. Management was conservative, and by the one-month follow-up, the patient’s ptosis and diplopia had resolved with recovery of ocular motility. This case illustrates a pupil-sparing partial oculomotor nerve palsy attributable to diabetic microangiopathy, which can mimic more ominous causes of third nerve palsy. It underscores the importance of recognizing the characteristic features of superior divisional third cranial nerve palsy while still promptly excluding compressive etiologies with appropriate imaging. With proper risk factor management, ischemic diabetic third nerve palsies carry an excellent prognosis, often with complete recovery within three months. Early diagnosis and conservative management in such cases lead to full neurological recovery and prevent unnecessary invasive interventions.

## Introduction

Oculomotor nerve (third cranial nerve) palsy is a well-recognized cause of ophthalmoplegia and ptosis. The oculomotor nerve innervates most of the extraocular muscles (superior rectus, medial rectus, inferior rectus, and inferior oblique) as well as the levator palpebrae superioris muscle of the upper eyelid. Anatomically, it divides into a superior division (supplying the superior rectus and levator palpebrae) and an inferior division (supplying the medial and inferior recti, inferior oblique, and carrying parasympathetic fibers to the pupil) at the anterior cavernous sinus or superior orbital fissure [1]. A complete third nerve palsy typically presents with “down and out” eye positioning, ptosis, and, depending on lesion location, pupil dilation [2].

Diabetes mellitus (DM) is among the most common causes of isolated cranial nerve palsies in adults, and third cranial nerve palsy is the second most frequent ocular motor diabetic neuropathy, with abducens nerve palsy occurring more commonly [3]. The pathophysiology in DM involves microvascular ischemia to the nerve’s vasa nervorum, leading to acute demyelinating injury of the somatic fibers while often preserving the superficial parasympathetic pupillomotor fibers [4]. The oculomotor nerve receives its vascular supply primarily through the vasa nervorum arising from the posterior cerebral and superior cerebellar arteries. Indeed, in patients over age 50, an isolated, pupil-sparing third nerve palsy is most often due to microvascular ischemia secondary to DM or hypertension. Such patients typically present with sudden-onset diplopia and ptosis, whereas the pupil remains reactive, distinguishing the condition from the ophthalmoplegia with mydriasis seen in compressive lesions [5].

Isolated involvement of only one division of the oculomotor nerve, however, is exceedingly rare. Most cases of divisional third nerve palsy reported in the literature are due to compressive or inflammatory lesions affecting the nerve branch selectively (e.g., aneurysms, cavernous sinus meningiomas, orbital trauma) [6-9]. An isolated superior division palsy is uncommon in a microvascular setting.

Hence, we present a case of a 59-year-old patient with DM and an uncommon, isolated superior division oculomotor palsy. This case report underscores the diagnostic challenge of such a partial nerve palsy and emphasizes the generally favorable prognosis of diabetic microvascular third nerve palsy, even in this atypical distribution.

## Case presentation

**Figure 1:**
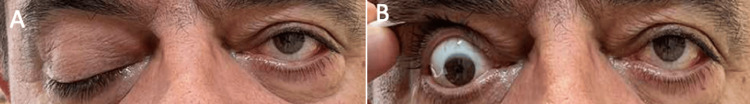
Blepharoptosis (A) and hypotropia (B) of the right eye in primary gaze position

A 59-year-old male presented to the emergency department with an acute onset of right-sided blepharoptosis and diplopia (Figure 1). The diplopia was binocular and was reported predominantly in primary gaze and in the vertical axis, worsening during upward gaze. The patient denied any headache, ocular pain, limb weakness, or other neurological symptoms. His medical history was significant for type 2 DM and dyslipidemia, managed with oral hypoglycemic agents and antilipidemic medication. He was also an active smoker (20 pack-year history). The patient reported no family history of cardiovascular, neurological, or autoimmune diseases and denied any history of prior operations. There was no history of recent trauma, and no similar episodes had occurred in the past.

**Figure 2:**
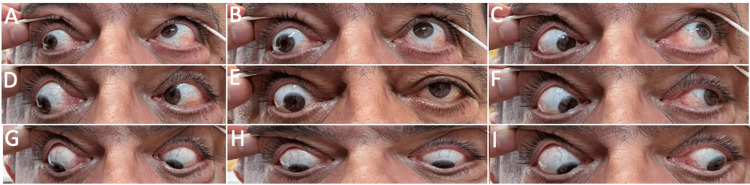
Nine positions of gaze at initial presentation (A-I). There is limitation of elevation of the right eye (A-C)

On examination, the patient was alert and oriented, with normal vital signs. Ophthalmologic evaluation revealed pupil-sparing right blepharoptosis, obscuring the visual axis, and limitation in upward mobility. On extraocular motility testing, the right eye had a marked limitation in upward gaze, while the rest of the ocular movements were intact. The left eye had a full range of motion in all directions (Figure 2). Importantly, the pupils were equal in size and briskly reactive to light, with no relative afferent pupillary defect (RAPD) noted. Best corrected visual acuity (BCVA) was 20/20 in each eye, and confrontational visual field testing was full bilaterally. Intraocular pressure measured 14 mmHg in both eyes at 11:00 AM. Slit lamp examination was performed using a standard biomicroscope with diffuse and focal slit illumination to assess the corneal integrity, anterior chamber depth, and presence of cells or flare. Moreover, fundus examination was conducted with indirect ophthalmoscopy using a 20D lens under iris dilation, supplemented by slit-lamp biomicroscopy with a 90D lens, and was unremarkable in both eyes.

Aside from the ocular findings, the rest of the neurologic exam was normal, as there were no motor or sensory deficits, and other cranial nerves were intact. Notably, there was no partial facial muscle weakness or fatigability of the ptosis during prolonged upgaze, which made a neuromuscular junction disorder less likely.

The clinical presentation raised suspicion for a microvascular ischemic cranial neuropathy. Nevertheless, given the potentially life-threatening differential diagnoses, a thorough evaluation was warranted. Compressive lesions, such as an aneurysm of the posterior communicating artery, intracranial tumors, stroke affecting the oculomotor nerve pathway, myasthenia gravis, which can cause fluctuating ptosis and ophthalmoparesis, and giant cell arteritis, which can cause ischemic cranial neuropathies in older adults, should be investigated. The absence of any pupillary dilation or subjective headache/ocular pain, as well as the intactness of other cranial nerves, made an aneurysmal compression or cavernous sinus syndrome less likely clinically. Myasthenia gravis was considered because of the ptosis and diplopia; however, the ptosis in this case was constant (not variable or fatigable), and the pattern of extraocular muscle involvement corresponded exactly to the superior division of cranial nerve III, rather than a diffuse or shifting weakness, making a primary nerve palsy more likely than a neuromuscular junction disorder. Additionally, thyroid function tests, including thyroid-stimulating hormone (TSH), free thyroxine (T4), and free triiodothyronine (T3), were performed, and all results were within normal limits.

A full laboratory evaluation was performed, including a complete blood count, metabolic panel, markers of systemic inflammation, such as erythrocyte sedimentation rate (ESR), and C-reactive protein (CRP). All laboratory parameters were within normal limits, except for an elevated glycated hemoglobin (HbA1c) of 7.1%, indicating suboptimal glycemic control and suggesting chronic hyperglycemia as a risk factor for microvascular complications. A non-contrast cranial computed tomography (CT) scan was performed during the initial assessment and revealed no acute hemorrhage, mass, or other abnormalities (Table 1).

**Table 1:** Laboratory blood tests AST: aspartate aminotransferase; ALT: alanine aminotransferase; γGT: gamma-glutamyl transferase; ALP: alkaline phosphatase; LDH: lactate dehydrogenase; CPK: creatine phosphokinase; CK-MB: creatine kinase-MB isoenzyme; TSH: thyroid-stimulating hormone; T4: thyroxine; T3: triiodothyronine; CRP: C-reactive protein; ANA: antinuclear antibody; RF: rheumatoid factor; RPR: rapid plasma reagin; HbA1c: glycated hemoglobin

Laboratory test	Value	Reference range
White blood cells	7.47 × 10^3^ /μL	4-11 × 10^3^ /μL
Neutrophils	65.6%	40-75%
Lymphocytes	25.8%	20-45%
Monocytes	7%	2-10%
Eosinophils	1.2%	1-6%
Basophils	0.4%	0.2-1%
Red blood cells	4.52 × 10^6^ /μL	3.8-6 × 10^6^ /μL
Hemoglobin	12.3 g/dL	11.8-17.8 g/dL
Hematocrit	39.6%	36-52%
Mean corpuscular volume	87.6 fL	80-96 fL
Mean corpuscular hemoglobin	27.2 pg	26-32 pg
Mean corpuscular hemoglobin concentration	31.1 pg/dL	32-36 pg/dL
Platelets	145 × 10^3 ^/μL	140-450 × 10^3 ^/μL
Erythrocyte sedimentation rate	25 mm	< 30 mm
Fasting blood sugar	100 mg/dL	70-115 mg/dL
HbA1c	7.1%	0-6%
Urea	41 mg/dL	0-50 mg/dL
Creatinine	1.09 md/dL	0.8-1.4 mg/dL
Potassium	4 mmol/dL	3.5-5.1 mmol/dL
Sodium	145 mmol/dL	136-146 mmol/dL
Magnesium	1.53 mEq/L	1.3-2.1 mEq/L
Calcium	9.2 mg/dL	8.2-10.5 mg/dL
Total proteins	6.2 g/dL	6.2-8.4 g/dL
Albumin	4.1 g/dL	3.5-5.1 g/dL
Total bilirubin	0.3 mg/dL	0.1-1.3 mg/dL
AST	22 IU/L	5-40 IU/L
ALT	33 IU/L	5-40 IU/L
γGT	21 IU/L	8-45 IU/L
ALP	69 IU/L	35-125 IU/L
LDH	213 IU/L	120-230 IU/L
CPK	110 IU/L	0-220 IU/L
CK-MB	13 IU/L	0-23 IU/L
Amylase	34 IU/L	28-100 IU/L
Uric acid	5.5 mg/dL	3.6-7.8 mg/dL
TSH	1.92 μIU/mL	0.35-4.94 μIU/mL
Free T4	1.03 ng/dL	0.70-1.48 ng/dL
Free T3	107.6 ng/dL	64-152 ng/dL
CRP	0.53 mg/dL	0-0.8 mg/dL
ANA	Negative	
RF	Negative	
RPR	Negative	

Subsequently, a high-resolution magnetic resonance imaging (MRI) of the brain and orbits was obtained, including magnetic resonance angiography (MRA) sequences to evaluate the intracranial vasculature (particularly the posterior communicating artery and circle of Willis). The MRI and MRA showed no evidence of aneurysms, compressive lesions, infarcts, or demyelinating lesions. Specifically, there were no structural lesions along the course of the right oculomotor nerve from its nucleus in the midbrain through the cavernous sinus to the orbit. These negative imaging results effectively ruled out compressive and infiltrative causes of the oculomotor palsy.

After this thorough evaluation, the diagnosis of a right superior division oculomotor nerve palsy secondary to diabetic microvascular ischemia was made. The clinical course and risk factor profile were in line with a diabetic superior division oculomotor nerve palsy caused by focal ischemia to the nerve. It is well documented that such ischemic cranial neuropathies in diabetic patients generally resolve spontaneously over time, as the nerve recovers once perfusion is restored or collateral circulation develops. The underlying risk factors were addressed, and the patient was advised to achieve strict glycemic control, adjusting antidiabetic medications in coordination with his primary care physician, and was counseled on smoking cessation to improve vascular health. For the diplopia, no patching of the eye was suggested, since the patient did not complain about discomfort.

**Figure 3:**
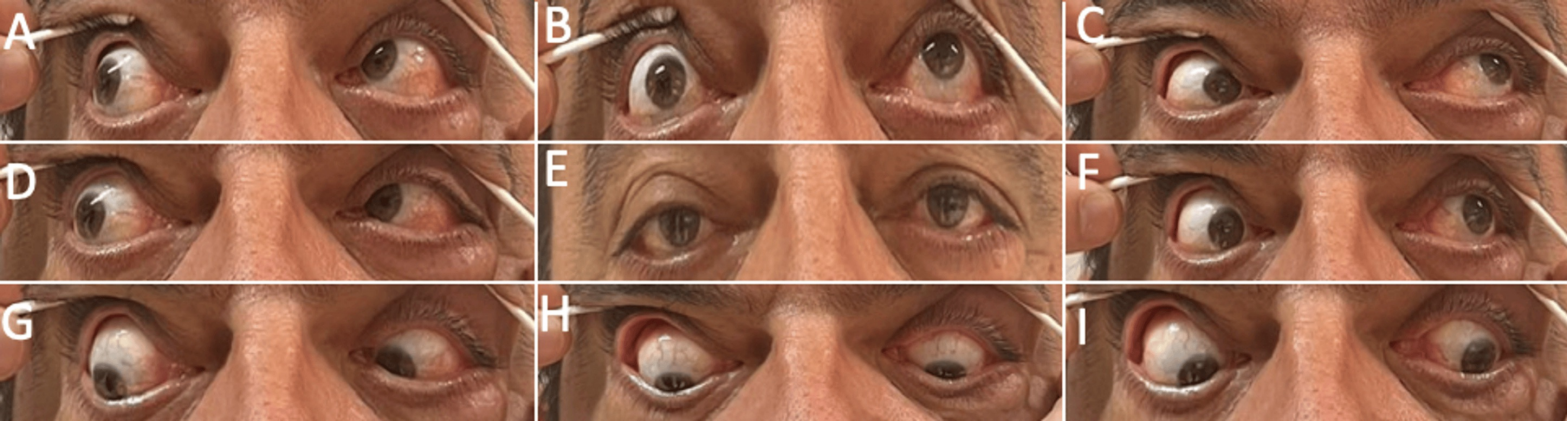
Nine positions of gaze (A-I) at one-month follow-up with significant improvement of his ophthalmologic deficits

At one-month follow-up, the patient demonstrated significant improvement of his ophthalmologic deficits. The right-sided ptosis had resolved, and his eyelid position was symmetric with normal levator function. Extraocular movement testing showed almost complete restoration of upward gaze in the right eye, and the patient no longer experienced any diplopia (Figure 3). This timely recovery is consistent with the expected course of diabetic oculomotor nerve palsy, where significant improvement typically occurs within weeks and complete recovery often within three months. By the time of this follow-up, his glycemic control had improved (repeat FBS = 82 mg/dL, HbA1c = 6.1%), and he reported significantly reducing his cigarette usage with intent to quit. No new neurological symptoms had developed. The patient’s outcome confirmed the initial clinical impression of an isolated ischemic neuropathy of the superior division of the oculomotor nerve. The patient continues to be monitored periodically, maintaining strict control of his DM and other vascular risk factors. Written informed consent was obtained from the patient for publication of the case details and accompanying images, and every effort has been made to ensure patient anonymity.

## Discussion

This case of a superior division oculomotor nerve palsy in a diabetic patient illustrates several important points in clinical neuro-ophthalmology. The patient’s presentation - acute onset blepharoptosis and limited elevation in one eye, with normal pupil function - is consistent with a partial third cranial nerve palsy localized to the superior branch. Such a presentation is distinctly rare in the absence of trauma or compressive lesions. In published reports, sudden isolated superior division palsy has often been linked to lesions near the cavernous sinus or orbital apex affecting the branch selectively [10]. However, DM and other microvascular risk factors can cause even a partial infarction of the oculomotor nerve, depending on which intraneural arterioles are compromised. In this patient, the pupil-sparing nature of the palsy strongly pointed toward a microvascular ischemic cause rather than an aneurysmal compression. Indeed, diabetic third nerve palsies classically spare the pupil because the ischemic damage predominantly involves the central somatic fibers, whereas the peripheral pupillomotor fibers remain intact.

Nonetheless, the unusual restriction to the superior division in our case prompted a thorough workup to exclude other causes, since an isolated branch palsy is not a routine diabetic manifestation. MRI and MRA of the brain and orbits were essential to rule out a compressive aneurysm of the posterior communicating artery and other intracranial lesions. In our patient, the normal imaging studies and the presence of DM as a risk factor supported the diagnosis of a diabetic superior division oculomotor palsy, a diagnosis of exclusion made after ruling out compressive/inflammatory etiologies, and led to conservative management.

The pathophysiology of diabetic oculomotor nerve palsy involves occlusion of the small penetrating arteries that supply the interior fibers of the nerve (vasa nervorum) due to hyperglycemia-induced vessel wall changes. Interestingly, there is a somatotopic organization within the oculomotor nerve, as fibers to different extraocular muscles occupy specific fascicular positions. Herein, in rare instances, a microvascular infarct leads to a superior division palsy, affecting the fascicles destined for the levator and superior rectus while sparing others. The superior division of the oculomotor nerve receives its vascular supply primarily through the vasa nervorum arising from small branches of the posterior cerebral and superior cerebellar arteries. Compared to the inferior division, the superior division has a relatively more robust and redundant vascular network, which may offer some protection against ischemic insults. This vascular resilience is thought to contribute to its lower susceptibility to microvascular cranial neuropathies [11].

Our case adds to the few reported instances and aligns with the hypothesis that the most interior portion of the oculomotor nerve can be selectively compromised by an ischemic lesion [12]. Myasthenia was clinically excluded, since no variability or fatigue effect was revealed, and midbrain lesions typically would not be so focal without other neurologic signs. Thus, the clinical reasoning favored a peripheral nerve ischemia after excluding alternative diagnoses.

A key aspect of this case is the prognosis. Diabetic (microvascular) third nerve palsies generally have an excellent outcome. Numerous studies have shown that ischemic cranial mononeuropathies tend to improve spontaneously over weeks to months as the nerve fiber conduction recovers. Recovery usually begins within the first 4-6 weeks, with significant improvement by three months in most patients. In fact, over 80% of microvascular third cranial nerve palsies completely resolve within about three months, and nearly all resolve by 6-12 months [13]. Our patient was managed conservatively with risk factor control, consistent with the standard approach for ischemic palsies. The expected course was explained, and indeed, as is typical, signs of recovery became evident within the first month. By that time, the patient’s ptosis and upgaze had markedly improved, reflecting remyelination and restoration of blood flow to the nerve. The absence of aberrant regeneration, such as eyelid retraction or pupil constriction on gaze movements, in diabetic third nerve palsy is another important clinical point - unlike compressive injuries that physically damage axons and often lead to miswiring during regeneration, microvascular palsies generally do not cause aberrant reinnervation [14]. In our case, there were no aberrant synkinesis phenomena during recovery, further supporting the ischemic etiology. If a third nerve palsy patient develops aberrant regeneration, a prior compressive insult must be suspected even if the initial presentation seemed of microvascular cause [15].

From management perspective, this case reinforces that observation with close follow-up is appropriate in diabetic pupil-sparing third nerve palsies, provided that neuroimaging has excluded intracranial lesions [13]. The prognosis is excellent, with a high likelihood of full recovery after about three months. During the palsy, supportive measures such as patching of one eye or prism glasses can help alleviate diplopia, which was not a problem in our case. Additionally, aggressive control of blood glucose and vascular risk factors was advised, as recurrent microvascular cranial neuropathies can occur if underlying risk factors remain uncontrolled [16].

## Conclusions

In conclusion, isolated superior division oculomotor nerve palsy is an uncommon presentation that poses a diagnostic challenge and may be caused by a life-threatening cause. This case highlights that diabetic microvascular neuropathy, albeit rarely, can produce a partial third cranial nerve palsy confined to the superior branch. It is imperative to exclude compressive or infiltrative causes in such partial palsies. Once a microvascular etiology is established, the outlook is very favorable. Diabetic third nerve palsies typically resolve over a few months with conservative management. Awareness of this expected recovery and the rarity of isolated division involvement can help prevent unnecessary interventions while ensuring that serious alternative diagnoses are not missed. This case adds to the literature on atypical diabetic cranial neuropathies and serves as a reminder that even unusual palsy patterns can occur in the setting of microvascular disease, fortunately with an excellent prognosis for functional recovery.
